# Collected mass spectrometry data on monoterpene indole alkaloids from natural product chemistry research

**DOI:** 10.1038/s41597-019-0028-3

**Published:** 2019-04-03

**Authors:** Alexander E. Fox Ramos, Pierre Le Pogam, Charlotte Fox Alcover, Elvis Otogo N’Nang, Gaëla Cauchie, Hazrina Hazni, Khalijah Awang, Dimitri Bréard, Antonio M. Echavarren, Michel Frédérich, Thomas Gaslonde, Marion Girardot, Raphaël Grougnet, Mariia S. Kirillova, Marina Kritsanida, Christelle Lémus, Anne-Marie Le Ray, Guy Lewin, Marc Litaudon, Lengo Mambu, Sylvie Michel, Fedor M. Miloserdov, Michael E. Muratore, Pascal Richomme-Peniguel, Fanny Roussi, Laurent Evanno, Erwan Poupon, Pierre Champy, Mehdi A. Beniddir

**Affiliations:** 1 Équipe “Pharmacognosie-Chimie des Substances Naturelles” BioCIS, Univ. Paris-Sud, CNRS, Université Paris-Saclay, 5 Rue J.-B. Clément, 92290 Châtenay-Malabry, France; 2 Centre for Natural Products and Drug Discovery, University of Malaya, Jalan Universiti, 50603 Kuala Lumpur, Wilayah Persekutuan, Kuala Lumpur, Malaysia; 3 EA921, SONAS, SFR QUASAV, UBL/Angers University, Campus du végétal, 42 rue Georges Morel, 49070 Beaucouzé, France; 4 Institute of Chemical Research of Catalonia (ICIQ), Barcelona Institute of Science and Technology, Avenue Països Catalans 16, 43007 Tarragona, Spain; 5 Departament de Química Organica i Analítica, Universitat Rovira i Virgili, C/Marcel `·lí Domingo s/n, 43007 Tarragona, Spain; 6 Laboratory of Pharmacognosy, CIRM, University of Liège, Quartier Hopital, 15 Avenue Hippocrate, Sart Tilman, 4000 Liège, Belgium; 7 Laboratoire de Pharmacognosie, UMR/CNRS 8638 COMETE, Faculté de Pharmacie de Paris, Université Paris Descartes, Sorbonne Paris Cité, 4 Avenue de l’Observatoire, 75006 Paris, France; 8 Laboratoire Écologie et Biologie des Interactions, Équipe Microbiologie de l’Eau, UMR CNRS 7267, Université de Poitiers, 5 rue Albert Turpain, 86073 Poitiers CEDEX 09, France; 9 Institut de Chimie des Substances Naturelles, CNRS-ICSN, UPR 2301, Université Paris-Saclay, 1 Avenue de la Terrasse, 91198 Gif-sur-Yvette, France; 10 Département de Pharmacognosie, Laboratoire PEIRENE-EA 7500, Faculté de Pharmacie, Université de Limoges, 2 rue du Dr Marcland, 87025 Limoges CEDEX, France

**Keywords:** Metabolomics, Data acquisition, Natural products

## Abstract

This Data Descriptor announces the submission to public repositories of the monoterpene indole alkaloid database (MIADB), a cumulative collection of 172 tandem mass spectrometry (MS/MS) spectra from multiple research projects conducted in eight natural product chemistry laboratories since the 1960s. All data have been annotated and organized to promote reuse by the community. Being a unique collection of these complex natural products, these data can be used to guide the dereplication and targeting of new related monoterpene indole alkaloids within complex mixtures when applying computer-based approaches, such as molecular networking. Each spectrum has its own accession number from CCMSLIB00004679916 to CCMSLIB00004680087 on the GNPS. The MIADB is available for download from MetaboLights under the identifier: MTBLS142 (https://www.ebi.ac.uk/metabolights/MTBLS142).

## Abstract (summary)

**Table:** 

Design Type(s)	mass spectrometry data transformation objective • mass spectrometry data analysis objective • data integration objective
Measurement Type(s)	mass spectrum
Technology Type(s)	liquid chromatography-tandem mass spectrometry
Factor Type(s)	
Sample Characteristic(s)	Strychnos usambarensis • Picralima nitida • Geissospermum laeve • Pleiocarpa mutica • Alstonia • Callichilia inaequalis • Chimarris cymosa • Mostuea brunonis • Gonioma < moth > • Cinchona • Catharanthus roseus • Voacanga grandifolia

Machine-accessible metadata file describing the reported data (ISA-Tab format)

## Background & Summary

Monoterpene indole alkaloids (MIAs) constitute a broad class of nitrogen-containing plant-derived natural products composed of more than 3000 members^1^. This natural product class is found in hundreds of plant species from the Apocynaceae, Loganiaceae, Rubiaceae, Icacinaceae, Nyssaceae, and Gelsemiaceae plant families. Throughout the six past decades, the structural intricacies and biological activities of these molecules have captured the interest of many researchers all over the world^2^. Examples of MIAs are the antimalarial drug of choice till the mid of the last century, quinine; the antihypertensive reserpine, and vincristine and vinblastine, which are used directly or as derivatives for the treatment of several cancer types. Recently, much effort was directed toward understanding and manipulating the underlying biosynthetic pathways of MIAs in order to engineer them in microorganisms to allow industrial production of medicinally relevant compounds^3–5^. Although a large amount of knowledge has been accumulated concerning the early steps^6–8^ and the assembly of key intermediates, many questions are still unanswered, and the discovery of new members of this family may illuminate unexpected enzymes involved in the biosynthesis of this intriguing group of natural products.

**Fig. 1:**
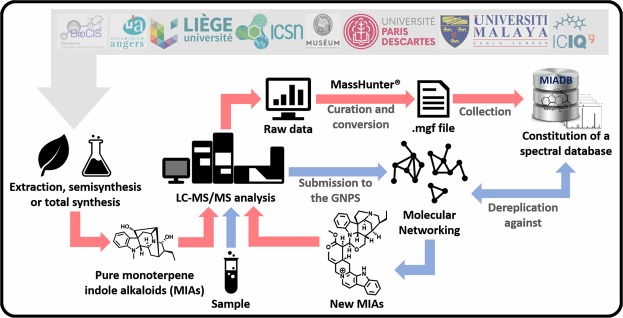
Construction of the MIADB (red arrows) and application in a molecular networking-based dereplication workflow (blue arrows).

As part of our continuing interest in MIA chemistry^9–12^, we developed a streamlined molecular networking^13^ dereplication pipeline based on the implementation of an in-house MS/MS database, constituted of a cumulative collection of MIAs^14^. In order to enrich this database, seven prominent practitioners from the global natural products research community shared their historical collections, leading to the construction of the largest MS/MS dataset of MIAs to date, that we named: Monoterpene Indole Alkaloids DataBase (MIADB) (Fig. 1). The MIADB contains MS/MS data of 172 standard compounds, comprising 128 monoindoles and 44 bisindoles (these compounds are presented in Supplementary Table 1) and covers more than 70% of the known (30/42) MIA skeletons. The information that can be drawn from this dataset is valuable for the scientific community that envisages the isolation of new MIAs.

The purpose of this Data Descriptor is to announce the deposition of the MIADB on the Global Natural Product Social Molecular Networking (GNPS^15^) and MetaboLights^16^. Each spectrum of the MIADB has its own accession number from CCMSLIB00004679916 to CCMSLIB00004680087 on GNPS (accessed via: https://gnps.ucsd.edu/ProteoSAFe/static/gnps-splash.jsp). The spectral collection is also is available for download from MetaboLights under the identifier: MTBLS142^17^.

## Methods

### Sample preparation

Each of the collected MIA was diluted to a concentration of 1 mg/mL using HPLC-grade (High Performance Liquid Chromatography) with MeOH (Methanol) as solvent. The solution was then transferred in 1.5 mL HPLC vials and analyzed by LC-MS/MS (Liquid Chromatography-tandem Mass Spectrometry). Chemicals and solvents were purchased from Sigma-Aldrich.

### Data acquisition

Samples were analyzed using an Agilent LC-MS (Liquid Chromatography Mass Spectrometry) system composed of an Agilent 1260 Infinity HPLC coupled to an Agilent 6530 ESI-Q-TOF-MS (ElectroSpray Ionization Quadrupole Time of Flight Mass Spectrometry) operating in positive mode. A Sunfire® analytical C_18_ column (150 × 2.1 mm; i.d. 3.5 *μ*m, Waters) was used, with a flow rate of 250 *μ*L/min and a linear gradient from 5% B (A: H_2_O + 0.1% formic acid, B: MeOH) to 100% B over 30 min. The column temperature was maintained at 25 °C. ESI conditions were set with the capillary temperature at 320 °C, source voltage at 3.5 kV, and a sheath gas flow rate of 10 L/min. Injection volume was set at 5 µL. The mass spectrometer was operated in Extended Dynamic Range mode (2 GHz). The divert valve was set to waste for the first 3 min. There were four scan events: positive MS, window from *m*/*z* 100–1200, then three data-dependent MS/MS scans of the first, second, and third most intense ions from the first scan event.

MS/MS settings were: three fixed collision energies (30, 50, and 70 eV), default charge of 1, minimum intensity of 5000 counts, and isolation width of *m*/*z* 1.3. Purine C_5_H_4_N_4_ [M + H]^+^ ion (*m*/*z* 121.050873) and hexakis(*1 H*,1 *H*,3*H*-tetrafluoropropoxy)-phosphazene C_18_H_18_F_24_N_3_O_6_P_3_ [M + H]^+^ ion (*m*/*z* 922.009798) were used as internal lock masses. Full scans were acquired at a resolution of 11 000 (at *m*/*z* 922) and 4000 at (*m/z* 121). A permanent MS/MS exclusion list criterion was set to prevent oversampling of the internal calibrant.

### Database constitution

The analysis of each of these substances resulted in 172 files with the standard.d format (Agilent standard data-format). A list of individual compounds for each sample was generated from an Auto MS/MS data mining process implemented in MassHunter® software on every single file. Averaged as well as monocollisional energy MS/MS spectra were generated from the three retained collision energies (30, 50, and 70 eV). Within this list, the molecular formula (as well as the exact mass) of the expected compound (in its charged state) was identified. Then, depuration of the other features was carried out. Finally, each spectrum was converted into the.mgf (Mascot Generic Format) using the export tool of the MassHunter® software.

## Data Records

All data described in this article have been uploaded to GNPS and MetaboLights. Each spectrum of the 172 compounds of the MIADB has its own accession number from CCMSLIB00004679916 to CCMSLIB00004680087 on the Global Natural Product Social Molecular Networking (GNPS) (accessed via: https://gnps.ucsd.edu/ProteoSAFe/static/gnps-splash.jsp). The spectral collection in its two versions (*i.e*. averaged and separate collision energy MS/MS spectra at 30, 50, and 70 eV) is available for download from MetaboLights under the identifier: MTBLS142^17^.

### Metadata

The MS/MS spectra of the MIADB library are recorded with a variety of details including: LC-MS/MS acquisition parameters, instrument details, organism, organism part, smiles and Inchi codes, CAS numbers, CHEBI IDs, retention times, and chemical formula. These metadata are available on the GNPS and MetaboLights websites.

## Technical Validation

### Spectroscopic validation of MIADB compounds

The structural identity of the alkaloids being implemented in the MIADB reference metabolite index was established through extensive spectroscopic analyses, including, NMR (Nuclear Magnetic Resonance) and HRMS (High-Resolution Mass Spectrometry). The analyses were carried out by the various collaborators having contributed to the establishment of the database. The obtained mass spectra were individually inspected to verify the occurrence of either the protonated molecular or molecular ion as the precursor mass.

### Selected strategies for the validation of the MIADB

The validation of the MIADB was achieved following two strategies: (i) dereplication of the profiled compounds from a methanol extract of the leaves of *Catharanthus roseus* (L.) G. Don. (Apocynaceae) (see supplementary Tables 2 and 3), and (ii) the dereplication of the MIADB against the MIAs previously available on the GNPS library before the upload of the MIADB.

### Molecular networking-based dereplication of *Catharanthus roseus* methanol extract

**Fig. 2:**
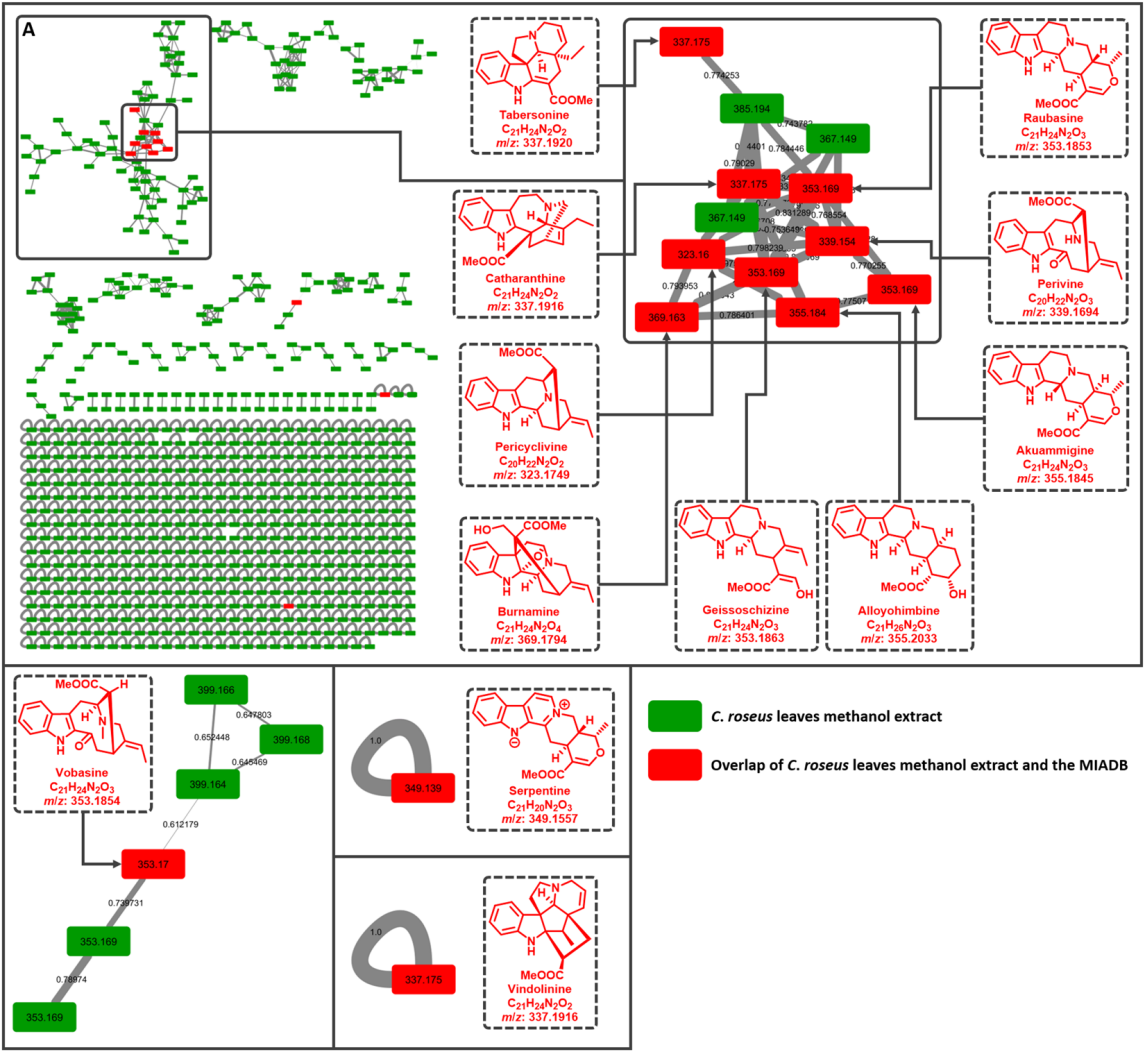
Full molecular network of the profiled compounds from a methanol extract of *C. roseus* leaves annotated by the MIADB. The cosine similarity score cutoff for the molecular network was set at 0.6, the parent ion mass tolerance at 0.02, the fragment ion mass tolerance at 0.02, the score library threshold at 0.6 and the minimum matched peaks at 6. The cosine similarity score are depicted on the edges.

Molecular networking-based dereplication using MIADB-uploaded GNPS libraries was attempted on the methanol extract of *Catharanthus roseus*, the MIAs content of which was thoroughly studied. Accordingly, more than 130 different compounds were reported from the different tissues of the plant^18^. In the displayed network, the experimental data of *C. roseus* methanol extract are depicted as green rectangles and nodes representing a consensus of experimental data and database records (*i.e*., MIADB-uploaded in the GNPS libraries) are displayed as red rectangles (Fig. 2). As expected, molecular networking of the *C. roseus* leaves methanol extract allowed dereplication of previously known metabolites within this plant including: tabersonine, catharanthine, vindolinine, perivine, geissoschizine, pericyclivine, serpentine, raubasine, and akuammigine (Table 1). All the dereplicated compounds were assigned a level of confidence 1 according to Schymanski *et al*.^19^ based on HMRS, MS/MS and retention time matching, except for geissoschizine, serpentine; and alloyohimbine. The latter were attributed a level of confidence of 2, due to a delta of retention time (RT) superior to 1.5 min. The molecular networking-based dereplication provided a comprehensive coverage of *C. roseus* alkaloids by regards to the available standards, despite the noticeable lack of a vinblastine hit. This missing observation is likely due to the vinblastine concentration that is known to be very low in the plant (ranging from 0.0003% to 0.001% w/w dry weight)^20^. Conversely, some unexpected matches could also be evidenced throughout the obtained dereplication: burnamine and vobasine. Although none of these were previously described in *C. roseus*, both these structural assignments can be deemed reasonable based on biosynthetic considerations. Being an akuammiline-derived MIA, such as akuammine^21^ and the monomer precursors of the bisindoles vingramine and methylvingramine^22^ that have been reported to occur in *C. roseus*, the detection of burnamine is not unexpected. Likewise, the co-dereplication in the depicted molecular network of the formerly described vobasane-type perivine supports the identification of vobasine within this plant. Such examples emphasize the dereplicative interest of MIADB especially on such a deeply dug plant model. Prior to its GNPS upload, *i.e*., as an in-house database, the ability of the MIADB to pinpoint tentatively new MIAs was demonstrated through the streamlined isolation of geissolaevine along with its *O*-methylether derivative and 3′,4′,5′,6′-tetrahydrogeissospermine from the formerly vastly studied *Geissospermum leave* (Vell.) Miers (Apocynaceae)^14^. Altogether, the currently garnered results support the valuable contribution of MIADB either for the straightforward identification of monoterpene indole alkaloids or to highlight putative structural novelty among this privileged structural class. The topology of the obtained network also reveals that a further extent of information could yet be accessed from *C. roseus* extracts. Indeed, most dereplicated MIAs are tightly associated within cluster A. Since clusterization depends on structural similarity, a single match to the MIADB-implemented GNPS allows for the propagation of the structure throughout an entire molecular family, indicating that most if not all the nodes of this cluster refer to MIAs. The seminal contribution of the MIADB to the tandem mass spectrometric databanks of MIA is expected to pave the way for the upload of such data by the numerous teams involved in MIA research all over the world, thereby contributing to making this tool more and more efficient to reach a quick and sharp insight into the MIAs content of any producing organism.

**Table 1:** Matches between the profiled compounds from a methanol extract of *C. roseus* and MIADB.

Compound	Match score	Comment	ΔRT (min)	Confidence level
**akuammigine**	0.69	Described in *C*. *roseus*	0.63	1
**alloyohimbine**	0.76	Not described in *C*. *roseus*	1.95	2
**burnamine**	0.62	Not described in *C*. *roseus*	1.07	1
**catharanthine**	0.67	Described in *C*. *roseus*	0.67	1
**geissoschizine**	0.71	Described in *C*. *roseus*	1.52	2
**pericyclivine**	0.79	Described in *C*. *roseus*	0.00	1
**perivine**	0.73	Described in *C*. *roseus*	0.01	1
**raubasine**	0.64	Described in *C*. *roseus*	0.26	1
**serpentine**	0.66	Described in *C*. *roseus*	6.65	2
**tabersonine**	0.80	Described in *C*. *roseus*	1.48	1
**vindolinine**	0.77	Described in *C*. *roseus*	1.44	1
**vobasine**	0.65	Not described in *C*. *roseus*	0.16	1

### Dereplication of the MIADB against the MIAs previously available on the GNPS library

As a second validation assay, the MIADB was dereplicated against the GNPS library. For this purpose, the 172.mgf files were submitted to the GNPS online platform and all the hits between the MIADB and the GNPS were annotated. 19 of the total MIAs were identified as hits by the GNPS platform (Table 2).

**Table 2:** MIADB matches with the GNPS library.

Compounds (GNPS)	Match score	Compounds (MIADB)	Comments
**brucine**	0.78	brucine	
**reserpiline**	0.86	reserpiline	
**tabernaemontanine**	0.74	tabernaemontanine	
**voachalotine**	0.93	voachalotine	
**ajmaline**	0.75	ajmaline	
**vincamine**	0.78	vincamine	
**methyl reserpate**	0.83	methyl reserpate	
**camptothecin**	0.73	camptothecin	
**reserpine**	0.86	reserpine	
**strychnine**	0.80	strychnine	
**akuammigine**	0.86	raubasine	epimer
**raubasine**	0.88	akuammigine	epimer
**corynanthine**	0.90	yohimbine	epimer
**yohimbine**	0.92	corynanthine	epimer
**vincosamide**	0.93	strictosamide	epimer
**strictosamide**	0.93	vincosamide	epimer
**yohimbine**	0.90	pseudoyohimbine	epimer
**elegantissine**	0.73	carapanaubine	isomer
**yohimbine**	0.89	alloyohimbine	epimer

These results indicate that the compounds from the 19 matches were correctly identified within the GNPS library, except in the case of epimers or isomers. Indeed, it should be noted that the matching process does not take into account the stereochemistry of the compounds **(**Table 2**)**.

## Supplementary Information

### ISA-Tab metadata file

Download metadata file

### Supplementary information

Supplementary Information

## Data Availability

The LC-MS feature detection software (MassHunter®) used in this work is commercially available from Agilent®.

## References

[1] Pan Q, Mustafa NR, Tang K, Choi YH, Verpoorte R (2016). Monoterpenoid indole alkaloids biosynthesis and its regulation in *Catharanthus roseus*: A literature review from genes to metabolites. Phytochemistry Rev.

[2] Pritchett BP, Stoltz BM (2018). Enantioselective palladium-catalyzed allylic alkylation reactions in the synthesis of *Aspidosperma* and structurally related monoterpene indole alkaloids. Nat. Prod. Rep..

[3] Qu Y (2015). Completion of the seven-step pathway from tabersonine to the anticancer drug precursor vindoline and its assembly in yeast. Proc. Natl. Acad. Sci. USA.

[4] Caputi L (2018). Missing enzymes in the biosynthesis of the anticancer drug vinblastine in Madagascar periwinkle. Science.

[5] Dang T-TT (2018). Sarpagan bridge enzyme has substrate-controlled cyclization and aromatization modes. Nat. Chem. Biol..

[6] Miettinen K (2014). The seco-iridoid pathway from *Catharanthus roseus*. Nat. Commun..

[7] Salim V, Yu F, Altarejos J, Luca V (2013). Virus-induced gene silencing identifies *Catharanthus roseus* 7-deoxyloganic acid-7-hydroxylase, a step in iridoid and monoterpene indole alkaloid biosynthesis. Plant J..

[8] Asada K (2013). A 7-deoxyloganetic acid glucosyltransferase contributes a key step in secologanin biosynthesis in Madagascar periwinkle. Plant Cell.

[9] Lachkar D (2017). Unified biomimetic assembly of voacalgine A and bipleiophylline via divergent oxidative couplings. Nat. Chem..

[10] Otogo N’Nang Obiang E (2017). Pleiokomenines A and B: Dimeric aspidofractinine alkaloids tethered with a methylene group. Org. Lett..

[11] Beniddir MA, Genta-Jouve G, Lewin G (2018). Resolving the (19*R*) absolute configuration of lanciferine, a monoterpene indole alkaloid from *Alstonia boulindaensis*. J. Nat. Prod..

[12] Otogo N’Nang E (2018). Theionbrunonines A and B: Dimeric vobasine alkaloids tethered by a thioether bridge from *Mostuea brunonis*. Org. Lett..

[13] Yang JY (2013). Molecular networking as a dereplication strategy. J. Nat. Prod..

[14] Fox Ramos AE (2017). Revisiting previously investigated plants: A molecular networking-based study of *Geissospermum laeve*. J. Nat. Prod..

[15] Wang M (2016). Sharing and community curation of mass spectrometry data with Global Natural Products Social Molecular Networking. Nat. Biotechnol..

[16] Haug K (2013). MetaboLights—an open-access general-purpose repository for metabolomics studies and associated meta-data. Nucl. Acids Res..

[17] Fox Ramos, A. E. *et al*. Collected mass spectrometry data on monoterpene indole alkaloids from natural product chemistry research. *MetaboLights*, http://identifiers.org/metabolights:MTBLS142 (2018). 10.1038/s41597-019-0028-3 PMC6480975 30944327

[18] Heijden RVD, Jacobs DI, Snoeijer W, Hallard D, Verpoorte R (2004). The *Catharanthus* alkaloids: Pharmacognosy and biotechnology. Curr. Med. Chem..

[19] Schymanski EL (2014). Identifying small molecules via high resolution mass spectrometry: Communicating confidence. Environment. Sci. Technol..

[20] Uniyal GC, Bala S, Mathur AK, Kulkarni RN (2001). Symmetry C18 column: a better choice for the analysis of indole alkaloids of *Catharanthus roseus*. Phytochem. Anal.

[21] Ramirez A, Garcia-Rubio S (2003). Current progress in the chemistry and pharmacology of akuammiline alkaloids. Curr. Med. Chem..

[22] Jossang A, Fodor P, Bodo B (1998). A new structural class of bisindole alkaloids from the seeds of *Catharanthus roseus*: Vingramine and methylvingramine. J. Org. Chem..

