# Supplementary material for: Collected mass spectrometry data on monoterpene indole alkaloids from natural product chemistry research
Source: Sci Data. 2019 Apr 3;6:15. doi: 10.1038/s41597-019-0028-3 (PMC6480975; doi:10.1038/s41597-019-0028-3)
Supplement: Supplementary file 2 — Supplementary Information [file 41597_2019_28_MOESM2_ESM.docx]

**Supplementary Information**

**Collected mass spectrometry data on monoterpene indole alkaloids from natural product chemistry research**

**Authors**

Alexander E. Fox Ramos^1^, Pierre Le Pogam^1^, Charlotte Fox Alcover^1^, Elvis Otogo N'Nang^1^, Gaëla Cauchie^1^, Hazrina Hazni^1,2^, Khalijah Awang^2^, Dimitri Bréard^3^, Antonio M. Echavarren,^4,5^ Michel Frederich^6^, Thomas Gaslonde^7^, Marion Girardot^8^, Raphaël Grougnet^7^, Mariia S. Kirillova,^4^ Marina Kritsanida^7^, Christelle Lémus,^7^ Anne-Marie Le Ray^3^, Guy Lewin^1^, Marc Litaudon^9^, Lengo Mambu^10^, Sylvie Michel^7^, Fedor M. Miloserdov^4^, Michael E. Muratore^4^, Pascal Richomme-Peniguel^3^, Fanny Roussi^9^, Laurent Evanno^1^, Erwan Poupon^1^, Pierre Champy^1^ & Mehdi A. Beniddir^1^

**Affiliations**

1. Équipe “Pharmacognosie-Chimie des Substances Naturelles” BioCIS, Univ. Paris-Sud, CNRS, Université Paris-Saclay, 5 Rue J.-B. Clément, 92290 Châtenay-Malabry, France.

2. Centre for Natural Products and Drug Discovery, University of Malaya, Jalan Universiti, 50603 Kuala Lumpur, Wilayah Persekutuan Kuala Lumpur, Malaysia.

3. EA921, SONAS, SFR QUASAV, UBL/Angers University, Campus du végétal, 42 rue Georges Morel, 49070 Beaucouzé, France.

4. Institute of Chemical Research of Catalonia (ICIQ), Barcelona Institute of Science and Technology, Avenue Països Catalans 16, 43007 Tarragona, Spain.

5. Departament de Química Orgànicà i Analítica, Universitat Rovira i Virgili, C/Marcel ̀ lí Domingo s/n, 43007 Tarragona, Spain.

6. Laboratory of Pharmacognosy, CIRM, University of Liège, Quartier Hopital, 15 Av. Hippocrate, Sart Tilman, 4000 Liège, Belgium.

7. Laboratoire de Pharmacognosie, UMR/CNRS 8638 COMETE, Faculté de Pharmacie de Paris, Université Paris Descartes, Sorbonne Paris Cité, 4 Avenue de l’Observatoire, 75006 Paris, France.

8. Laboratoire Écologie et Biologie des Interactions, Équipe Microbiologie de l’Eau, UMR CNRS 7267, Université de Poitiers, 5 rue Albert Turpain, 86073 Poitiers CEDEX 09, France.

9. Institut de Chimie des Substances Naturelles, CNRS-ICSN, UPR 2301, Université Paris-Saclay, 1 Avenue de la Terrasse, 91198 Gif-sur-Yvette, France.

10. Département de Pharmacognosie, Laboratoire PEIRENE-EA 7500, Faculté de Pharmacie, Université de Limoges, 2 rue du Dr Marcland, 87025 Limoges CEDEX, France.

Corresponding author: Mehdi A. Beniddir (mehdi.beniddir@u-psud.fr)

Table of Contents

[**Supplementary Table 1. Monoterpene indole alkaloids included in the MIADB** 3](#_Toc530646910)

[**First validation assay:** 30](#_Toc530646911)

[**Supplementary Table 2. Molecular Networking parameters for the first validation assay** 30](#_Toc530646912)

[**Second validation assay:** 30](#_Toc530646913)

[**Supplementary Table 3. Molecular Networking parameters for the second validation assay** 30](#_Toc530646914)

# **Supplementary Table 1. Monoterpene indole alkaloids included in the MIADB**

| Substance | Measured *m*/*z* | Source | Retention time (min) | Structure |
| --- | --- | --- | --- | --- |
| 10-Hydroxygeissoschizol | 313.1903 | Natural | 11.65 |  |
| 10-Hydroxyusambarine | 467.2822 | Natural | 13.60 |  |
| 11-Hydroxyusambarine | 467.2794 | Natural | 13.33 |  |
| 11-Methoxyyohimbine | 385.2125 | Natural | 14.08 |  |
| 14,15-Dehydrovincamine | 353.1859 | Natural | 12.98 |  |
| 18,19-Dihydrousambarine | 453.3021 | Natural | 14.74 |  |
| 18-Hydroxyisosungucine | 651.3325 | Natural | 15.35 |  |
| 19,20-Didehydroervatamine | 353.1860 | Natural | 14.26 |  |
| 19,20-Dihydrousambarensine | 435.2554 | Natural | 14.53 |  |
| 19,20-Epoxynovacine | 441.2012 | Natural | 10.05 |  |
| 2,7-Dihydroxyapogeissoschizine | 369.1818 | Natural | 16.49 |  |
| 3,4,5,6-Tetradehydrogeissoschizol | 293.1645 | Natural | 16.74 |  |
| 3',4',5',6'-Tetradehydrolongicaudatine Y | 567.3127 | Natural | 15.20 |  |
| 3′,4′,5′,6′-Tetradehydrogeissospermine | 629.3499 | Natural | 15.81 |  |
| 3-Epimeloscine | 293.1655 | Natural | 11.70 |  |
| 3'-Oxotabernaelegantine B | 721.3944 | Natural | 22.61 |  |
| 3-R,S-Hydroxytabernaelegantine A | 705.4009  [M+H-H_2_O]^+^ | Natural | 15.07 |  |
| 6,7-Dihydroflavopereirine | 249.1378 | Natural | 16.28 |  |
| Acetyl-splendoline | 413.2077 | Natural | 14.36 |  |
| Ajmaline | 327.2096 | Natural | 12.55 |  |
| Akagerine | 325.1930 | Natural | 15.89 |  |
| Akuammicine | 323.1770 | Natural | 16.07 |  |
| Akuammidine | 353.1864 | Natural | 11.11 |  |
| Akuammigine | 353.1843 | Natural | 18.14 |  |
| Akuammiline | 395.1969 | Natural | 13.52 |  |
| Akuammine | 383.1962 | Natural | 12.57 |  |
| Alloyohimbine | 355.2034 | Natural | 14.30 |  |
| Alpha-colubrine | 365.1861 | Natural | 13.80 |  |
| Alpha-methylyohimbine | 369.2181 | Natural | 16.76 |  |
| Angustine | 314.1281 | Natural | 28.84 |  |
| Antirhine | 297.1958 | Natural | 18.29 |  |
| Apparicine | 265.1687 | Natural | 18.50 |  |
| Aspidofractinine | 281.2012 | Natural | 9.26 |  |
| Bipleiophylline | 795.3385 | Semisynthesis | 19.49 |  |
| Brucine | 395.1967 | Natural | 12.95 |  |
| Burnamine | 369.1791 | Natural | 14.62 |  |
| Cadambine | 545.2161 | Natural | 16.74 |  |
| Camptothecine | 349.1174 | Commercial  (Alfa Aesar, 2016) | 27.21 |  |
| Carapanaubine | 429.2067 | Natural | 15.75 |  |
| Catharanthine | 337.1914 | Natural | 16.11 |  |
| Ceridimine | 497.2898 | Natural | 12.62 |  |
| C-fluorocurarine | 307.1808 | Natural | 11.29 |  |
| Cinchonamine | 297.1951 | Natural | 16.68 |  |
| C-mavacurine | 309.1965 | Natural | 14.82 |  |
| Condylocarpine *N*-oxide | 339.1719 | Natural | 15.35 |  |
| Conopharyngine | 399.2292 | Natural | 16.30 |  |
| Coronaridine | 339.2056 | Natural | 16.85 |  |
| Corynantheidal | 297.1950 | Natural | 14.43 |  |
| Corynantheidine | 369.2183 | Natural | 16.78 |  |
| Corynantheidol | 299.2120 | Natural | 14.05 |  |
| Corynantheine | 367.2006 | Natural | 16.70 |  |
| Corynantheol | 297.1963 | Natural | 13.66 |  |
| Corynanthine | 355.2011 | Natural | 14.25 |  |
| Criophylline | 647.3569 | Natural | 15.99 |  |
| Cymoside | 547.2286 | Natural | 10.35 |  |
| Desacetyl-isosplendine | 327.2073 | Natural | 15.95 |  |
| Desoxycabufiline | 735.4127 | Natural | 16.90 |  |
| Dregamine | 355.2012 | Natural | 11.90 |  |
| Echitamidine | 341.1868 | Natural | 10.85 |  |
| Echitamine | 385.2123 | Natural | 13.40 |  |
| Ervafoline | 645.3433 | Natural | 25.80 |  |
| Ervatamine | 355.2023 | Natural | 14.99 |  |
| Ervitsine | 293.1648 | Natural | 14.24 |  |
| Geissolaevine | 367.1298 | Natural | 23.22 |  |
| Geissolosimine | 573.3609 | Natural | 13.83 |  |
| Geissoschizine | 353.1869 | Natural | 18.31 |  |
| Geissoschizoline | 299.2114 | Natural | 10.36 |  |
| Geissospermine | 633.3801 | Natural | 14.42 |  |
| Gelsemicine | 359.1964 | Natural | 16.28 |  |
| Gelsemine | 323.1769 | Natural | 9.75 |  |
| Goniomedine A | 649.3741 | Natural | 12.49 |  |
| Goniomedine B | 633.3790 | Natural | 14.66 |  |
| Goniomedinone | 663.3532 | Natural | 13.83 |  |
| Goniomedine A *N*-oxide | 665.3705 | Natural | 13.40 |  |
| Goniomitine | 299.2104 | Natural | 17.49 |  |
| Grandilodine B | 455.1821 | Total synthesis | 25.06 |  |
| Grandilodine C | 381.1448 | Total synthesis | 24.09 |  |
| Holstiine | 383.1976 | Natural | 12.06 |  |
| Holstiline | 397.2141 | Natural | 16.00 |  |
| Ibogaine | 311.2118 | Natural | 13.57 |  |
| Ibogamine | 281.1998 | Natural | 15.34 |  |
| Iboxygaine | 327.2077 | Natural | 14.90 |  |
| Icajine | 365.1866 | Natural | 13.64 |  |
| Isomalindine | 304.1802 | Natural | 10.88 |  |
| Isoretuline | 339.2057 | Natural | 12.55 |  |
| Isostrychnine | 335.1741 | Natural | 12.14 |  |
| Isostrychnopentamine A | 550.3544 | Natural | 13.16 |  |
| Isosungucine | 635.3363 | Natural | 16.03 |  |
| Lanciferine | 545.2268 | Natural | 29.88 |  |
| Leuconolam | 327.1702 | Natural | 18.12 |  |
| Leucoridine A | 557.3640 | Semisynthesis | 16.27 |  |
| Lundurine A | 367.1652 | Total synthesis | 28.08 |  |
| Malindine | 304.1819 | Natural | 10.20 |  |
| Melosuavine E | 703.3472 | Natural | 16.70 |  |
| Methuenine | 295.1804 | Natural | 14.35 |  |
| Methyle reserpate | 415.2234 | Natural | 16.12 |  |
| Mitrinermine | 385.2141 | Natural | 16.43 |  |
| *N*^4^-methylantirhine | 311.2123 | Natural | 14.23 |  |
| Naucleidinal | 337.1543 | Natural | 27.33 |  |
| Naulafine | 312.1124 | Natural | 22.14 |  |
| *N*^b^-methylusambarensine | 447.2546 | Natural | 15.48 |  |
| Novacine | 425.2065 | Natural | 13.05 |  |
| Ochrolifuanine A | 439.2850 | Natural | 16.62 |  |
| Ochropamine | 367.2021 | Natural | 16.30 |  |
| Ochropposinine | 359.2310 | Natural | 16.60 |  |
| Olivacine | 247.1228 | Natural | 21.48 |  |
| *O*-methylgeissolaevine | 381.1446 | Natural | 26.97 |  |
| *O*-trimethoxy-3,4,5-benzoyl-OH-vincamajine | 577.255 | Natural | 15.76 |  |
| O-3,4,5-trimethoxycinnamate-OH-vincamajine | 603.2701 | Natural | 21.00 |  |
| Panarine | 323.1754 | Natural | 15.64 |  |
| Pandine | 353.1843 | Natural | 14.82 |  |
| Pericyclivine | 323.1749 | Natural | 16.17 |  |
| Perivine | 339.1694 | Natural | 13.40 |  |
| Picraline | 411.1914 | Natural | 17.32 |  |
| Pleiocarpamine | 323.1742 | Natural | 17.46 |  |
| Pleiocarpine | 397.2122 | Natural | 26.94 |  |
| Pleiocarpinilam | 367.2032 | Natural | 15.06 |  |
| Pleiocarpinine | 353.2217 | Natural | 17.23 |  |
| Pleiocarpoline | 413.2088 | Natural | 17.16 |  |
| Pleiokomenine A | 717.4375 | Natural | 19.52 |  |
| Pleiokomenine B | 689.4051 | Natural | 17.96 |  |
| Pleiomutine | 631.4026 | Natural | 18.73 |  |
| Pleiomutinine | 615.3667 | Natural | 15.01 |  |
| Polyneuridine | 353.1874 | Natural | 12.95 |  |
| Protostrychnine | 353.1865 | Natural | 10.05 |  |
| Pseudostrychnine | 351.1686 | Natural | 13.27 |  |
| Pseudoyohimbine | 355.2030 | Natural | 13.63 |  |
| Quebrachamine | 283.2185 | Natural | 15.05 |  |
| Quebrachidine | 353.1861 | Natural | 14.91 |  |
| Quinidine | 325.1907 | Natural | 13.69 |  |
| Raubasine | 353.1849 | Natural | 17.54 |  |
| Rauvomitine | 503.2531 | Natural | 22.61 |  |
| Reserpiline | 413.2077 | Natural | 17.02 |  |
| Reserpine | 609.2817 | Natural | 21.85 |  |
| Retuline | 339.2063 | Natural | 9.59 |  |
| Serpentine | 349.1557 | Natural | 13.15 |  |
| 20'-episerpentinine | 685.3381 | Natural | 18.20 |  |
| Splendoline | 371.1973 | Natural | 11.85 |  |
| Strictosamide | 499.2071 | Natural | 26.66 |  |
| Strictosidine | 531.2330 | Natural | 16.96 |  |
| Strychnine | 335.1760 | Natural | 11.00 |  |
| Strychnofoline | 483.2757 | Natural | 11.94 |  |
| Strychnogucine A | 651.3347 | Natural | 15.55 |  |
| Strychnogucine C | 651.3361 | Natural | 16.33 |  |
| Strychnohexamine | 869.4934 | Natural | 17.81 |  |
| Strychnopentamine | 550.3547 | Natural | 12.58 |  |
| Strychnophylline | 566.3494 | Natural | 8.71 |  |
| Sungucine | 635.3390 | Natural | 15.32 |  |
| Tabernaemontanine | 355.2008 | Natural | 14.73 |  |
| Tabernamine | 617.3835 | Natural | 15.28 |  |
| Tabernanthine | 311.2109 | Natural | 18.63 |  |
| Tabersonine | 337.1928 | Natural | 18.23 |  |
| Trimethoxy-3,4,5-cinnamate-vincamajine | 587.2748 | Natural | 17.77 |  |
| Tubotaiwine | 325.1897 | Natural | 13.70 |  |
| 10-hydroxyusambarensine | 449.2337 | Natural | 16.64 |  |
| Usambarine | 451.2857 | Natural | 14.80 |  |
| Vellosimine | 293.1651 | Natural | 14.71 |  |
| Villalstonine | 661.3737 | Natural | 17.38 |  |
| Vinblastine | 811.4268 | Natural | 18.62 |  |
| Vincamajine | 367.2013 | Natural | 12.01 |  |
| Vincamine | 355.2019 | Natural | 19.99 |  |
| Vincosamide | 499.2101 | Natural | 26.86 |  |
| Vindolinine | 337.1916 | Natural | 13.46 |  |
| Voacalgine A | 475.1853 | Natural | 19.64 |  |
| Voacamine | 705.3984 | Natural | 17.52 |  |
| Voacangine | 369.2179 | Natural | 16.76 |  |
| Voachalotine | 367.2033 | Natural | 14.25 |  |
| Vobasine | 353.1854 | Natural | 15.32 |  |
| Vobtusine | 719.3819 | Natural | 23.26 |  |
| Vomicine | 381.1809 | Natural | 13.39 |  |
| Wieland-Gumlich aldehyde | 311.1758 | Natural | 9.62 |  |
| Yohimbine | 355.2014 | Natural | 15.20 |  |

# **First validation assay:**

The plant material was a commercial batch of the leaves of *Catharanthus roseus* (L.) G.Don. used for teaching purposes (Univ. Paris-Sud), bearing no voucher number.

It was extracted as follows:

For LC-MS/MS analysis, 1 g of dry and powdered leaves of *C. roseus* were extracted with MeOH (20 mL). The MeOH crude extract was filtered under vacuum and concentrated by rotatory evaporation to yield 10 mg of residue. This residue was analyzed in identical conditions to that used for data acquisition of individual compounds.

# **Supplementary Table 2. Molecular Networking parameters for the first validation assay**

| Parameter | Value |
| --- | --- |
| Minimum pairs cosine | 0.6 |
| Parent mass ion tolerance | 0.02 |
| Fragment ion mass tolerance | 0.02 |
| Minimum matched peaks | 6 |
| Top K | 10 |
| Minimum cluster size | 2 |
| Maximum connected component size | 100 |
| Run MScluster | on |
| Library search score threshold | 0.6 |
| Library search minimum matched peaks | 6 |

A molecular network was created using the online workflow at GNPS. The data was then clustered with MS-Cluster with a parent mass tolerance of 0.02 Da and a MS/MS fragment ion tolerance of 0.02 Da to create consensus spectra. Further, consensus spectra that contained less than 2 spectra were discarded. A network was then created where edges were filtered to have a cosine score above 0.6 and more than 6 matched peaks. Further edges between two nodes were kept in the network if and only if each of the nodes appeared in each other's respective top 10 most similar nodes. The spectra in the network were then searched against GNPS' spectral libraries. All matches kept between network spectra and library spectra were required to have a score above 0.6 and at least 6 matched peaks.

# **Second validation assay:**

# **Supplementary Table 3. Molecular Networking parameters for the second validation assay**

| Parameter | Value |
| --- | --- |
| Minimum pairs cosine | 0.6 |
| Parent mass ion tolerance | 0.02 |
| Fragment ion mass tolerance | 0.02 |
| Minimum matched peaks | 6 |
| Top K | 10 |
| Minimum cluster size | 1 |
| Maximum connected component size | 100 |
| Run MScluster | off |
| Library search score threshold | 0.7 |
| Library search minimum matched peaks | 6 |

A molecular network was created using the online workflow at GNPS. The data was filtered by removing all MS/MS peaks within +/- 17 Da of the precursor m/z. MS/MS spectra were window filtered by choosing only the top 6 peaks in the +/- 50Da window throughout the spectrum. A network was then created where edges were filtered to have a cosine score above 0.6 and more than 6 matched peaks. Further edges between two nodes were kept in the network if and only if each of the nodes appeared in each other's respective top 10 most similar nodes. The spectra in the network were then searched against GNPS' spectral libraries. The library spectra were filtered in the same manner as the input data. All matches kept between network spectra and library spectra were required to have a score above 0.7 and at least 6 matched peaks.
